# Copeptin, Procalcitonin and Routine Inflammatory Markers–Predictors of Infection after Stroke

**DOI:** 10.1371/journal.pone.0048309

**Published:** 2012-10-31

**Authors:** Felix Fluri, Nils G. Morgenthaler, Beat Mueller, Mirjam Christ-Crain, Mira Katan

**Affiliations:** 1 Department of Neurology, University Hospital Basel, Basel, Switzerland; 2 Department of Neurology, University Hospital Zürich, Zürich, Switzerland; 3 Institut für Experimentelle Endokrinologie, Charité, Berlin, Germany; 4 Medical University Clinic, Cantonal Hospital Aarau, Aarau, Switzerland; 5 Department of Endocrinology, University Hospital Basel, Basel, Switzerland; 6 Columbia University, Department of Neurology, Division of Stroke, New York, New York, United States of America; University of Queensland, Australia

## Abstract

**Background:**

Early predictors for the development of stroke-associated infection may identify patients at high risk and reduce post-stroke infection and mortality.

**Methods:**

In 383 prospectively enrolled acute stroke patients we assessed time point and type of post-stroke infections (i.e. pneumonia, urinary tract infection (UTI) other infection (OI)). Blood samples were collected on admission, and days 1, and 3 to assess white blood cells (WBC), monocytes, C-reactive protein (CRP), procalcitonin (PCT), and copeptin. To determine the magnitude of association with the development of infections, odds ratios (OR) were calculated for each prognostic blood marker. The discriminatory ability of different predictors was assessed, by calculating area under the receiver operating characteristic curves (AUC). Prognostic models including the three parameters with the best performance were identified.

**Results:**

Of 383 patients, 66 (17.2%) developed an infection after onset of stroke. WBC, CRP, copeptin and PCT were all independent predictors of any infection, pneumonia and UTI developed at least 24 hours after measurements. The combination of the biomarkers WBC, CRP and copeptin (AUC: 0.92) and WBC, CRP and PCT (AUC: 0.90) showed a better predictive accuracy concerning the development of pneumonia during hospitalization compared to each marker by itself (p-Wald <0.0001).

**Conclusion:**

Among ischemic stroke patients, copeptin, PCT, WBC and CRP measured on admission were predictors of infection in general, and specifically for pneumonia and UTI within 5 days after stroke. The combination of these biomarkers improved the prediction of patients who developed an infection.

## Introduction

Infection during the first days after ischemic stroke (IS) occurs in 25–65% of patients [1], [2]. Pneumonia and urinary tract infection (UTI) are the most common infectious complications after IS [3]. It has been suggested that the predominance of infections during the acute phase of stroke [1] is due to stroke-induced immunosuppression (SIS) [4]. The central nervous system modulates the activity of the immune system through complex pathways that include the hypothalamic pituitary adrenal axis (HPAA), the vagus nerve, and the sympathetic nervous system [5], [6]. Several studies found an independent association between stroke-associated infections (SAI) and poor functional outcome after IS [7]–[9].

Therefore, early initiation of antibiotic treatments is recommended if infection is present [10]. However, gold-standard clinical diagnostics are time-consuming and delay early antibiotic therapy. Thus, accurate and simply available prognostic markers for optimal risk stratification are needed. We therefore selected C-reactive protein (CRP), white blood cells (WBC), monocytes (Mcyt), as they represent the most commonly measured and well-established inflammatory markers in clinical routine. Procalcitonin (PCT) was selected to better discriminate infections from general inflammation [11], [12]. Copeptin, a reliable stress marker [13] was selected because SIS may be mediated by changes in the neuroendocrine system. All these biomarkers are available immediately due to rapid analytic procedure.

We hypothesize that these blood markers are predictive for the development of post-stroke infections. First we planned to evaluate the prognostic value of each blood biomarker to predict infections in the acute phase of IS. Second, we aimed to identify the best prognostic model consisting of a batch of the best prognostic biomarkers. Thereafter, the prognostic value of this batch was compared to that of each prognostic biomarker alone.

## Patients and Methods

### Ethics Statement

The study has been approved by the local Ethics Committee at the University Hospital of Basel. All participants or their representative gave written informed consent for the study.

### Study Population

We performed a post-hoc analysis of a prospective cohort study [14]. All patients with IS within 72 hours before admission at the Emergency Department, University Hospital of Basel, were eligible and prospectively enrolled (11/2006–11/2007). IS was confirmed by CT and/or MRI on admission. Neurological deficits were measured at presentation with the National Institutes of Health Stroke Scale (NIHSS) score.

### Definition of Stroke-associated Infections

SAI was defined as any infection occurring within the first 5 days of hospital admission [13]. Infections were diagnosed according to the criteria of the U.S. Centers for Disease Control and Prevention (CDC) [15]. We distinguished between pneumonia, urinary tract infection (UIT) and “other infections” (OI). Pneumonia was diagnosed when at least one of each of the first and latter criteria was fulfilled: i) abnormal respiratory examination, pulmonary infiltrates in chest x-rays; ii) productive cough with purulent sputum, positive microbiological cultures from lower respiratory tract or blood cultures. Diagnosis of UTI was based on two of the following criteria: fever (≥38.0°C), urine sample positive for nitrite, leukocyturia (>40/µL), or significant bacteriuria (≥10^4^/mL of an uropathogen). OI was defined if temperature was ≥38.0°C, white blood cell count was ≥11000/mL or CRP≥10 mg/L and an infectious manifestation was present. Diagnosis of infection was done by the treating physician during hospitalization and was then validated post-hoc using charts, both diagnosis by treating physicians as well as secondary validation was blinded to biomarker levels with the exception of WBC and CRP for the diagnosis of (OI). Time point of diagnosis was referred to the beginning of clinical symptoms, which lead to diagnostic work-up and resulted in the diagnosis of infection.

In order to exclude acute infections preceding stroke, patients with admission temperature ≥38°C, or patients reporting an infection lasting up to 3 days before onset of stroke or patients who required mechanical intubation were not included in the study.

### Laboratory Methods

Blood samples were collected on admission (baseline) within 72 hours from symptom onset, and 1, and 3 days after admission to assess WBC and Mcyt count, CRP level, PCT and copeptin. PCT serum concentration was measured using a commercially available time-resolved amplified cryptate emission technology assay (Kryptor PCT, Brahms, Hennigsdorf, Germany) [16]. Measurement of copeptin was performed in a single batch with a commercial sandwich immunoluminometric assay (LUMItest CT-proAVP, B.R.A.H.M.S, Hennigsdorf/Berlin, Germany) [17]. In patients who died within 5 days after admission, or in patients who were discharged before day 5, only data from admission or until the day of discharge were collected.

### Statistical Analysis

Descriptive statistics were expressed as means ± standard deviations, medians and quartiles or absolute and relative frequencies depending on their distribution. Group differences were assessed using the Kruskal-Wallis test or Chi 2-test. Logarithmic transformation was performed to obtain an approximately normal distribution for all parameters except temperature and Mcyt.

First, the association of the biomarkers measured at admission with the presences of infections developed within 5 days was assessed using simple logistic regression.

Second we calculated pooled logistic regression considering patients to be at risk until the manifestation of an infection or until day 5 whichever occurred first. Each of these models had one time dependent predictor variable, i.e., the measurement of a given blood parameter 1 or 2 days before the respective day of diagnosis of infection. To adjust for potential clustering of data within subjects, robust standard errors were computed using the method of Huber-White. Odds ratios (OR) and associated 95% confidence intervals (95%CI) refer to an increase of the respective parameter from the lowest to the highest quartile.

Third, we compared the discriminatory ability of different predictors by calculating receiver operating characteristic (ROC) analysis. Bootstrap methods were used to derive 95%CIs for AUCs, index of Youden and optimal cutpoints to statistically compare AUC’s of different predictors.

Forth, to assess the prognostic independence from age, NIHSS score (as indicator of stroke severity) and Charlson index (as indicator of comorbidity burden) as well as infratentorial and supratentorial infarct localization, we performed bivariate logistic regression (to avoid over-fitting) with these potential confounders.

Finally, we calculated 2 prediction models (batch 1 and 2) by including established inflammatory parameters (WBC and CRP) and either Copeptin or PCT, the 2 new makers. Since robust precision estimates were used, model comparisons could not be done using likelihood ratio tests but were based on Wald p-values.

P-values less than 0.05 were considered to indicate statistical significance. All calculations were performed using SAS software, version 9.2 (SAS Institute Inc., Cary, NC, USA).

## Results

### Baseline Data

Of 383 patients with stroke, 66 (17.2%) developed an infection within 5 days after onset of stroke. Twenty (5.2%) patients suffered from pneumonia, 25 (6.5%) patients had UTI and 21 (5.5%) patients an OI (sepsis: 7 patients; phlebitis: 6 patients; gastroenteritis: 4 patients, erysipelas: 1 patient; panniculitis: 1 patient, colpitis: 2 patients). Baseline data are summarized in table 1.

**Table 1:** Baseline Data.

	All patients	Patients without infection	Patients with any infection	Pneumonia	UTI	Other infections
**N**	383	317	66	20	25	21
**Age**						
Median (±SD)	71.4±13.7	70.5±14.1	75.6±10.6	77.0±10.5	77.3±10.8	74.4
**Gender (male)**						
% (n)	57.7 (221)	60.8 (192)	43.3 (29)	45.0 (9)	32.0 (8)	50 (13)
**Laboratory Findings on admission**						
**CRP (mg/ml)**						
median	3.0	3.0	5.1	5.6	4.9	4.5
(IQR)	(3.0–6.7)	(3.0–5.8)	(3.0–15.8)	(3.0–19.7)	(3.0–24.3)	(3.0–8.8)
**WBC (10^9^/l)**						
median	8.0	7.8	9.7	9.8	9.9	9.2
(IQR)	(6.6–9.8)	(6.5–9.4)	(7.5–11.4)	(7.5–13.5)	(8.3–11.2)	(7.4–11.3)
**Monocyte (10^9^/l)**						
Mean (±SD)	0.410±0.167	0.398±0.143	0.463±0.243	0.557±0.357	0.471±0.277	0.413±0.152
**Procalcitonin (µg/l)**						
median	0.017	0.016	0.018	0.022	0.017	0.027
(IQR)	(0.01–0.02)	(0.01–0.02)	(0.01–0.03)	(0.02–0.03)	(0.01–0.04)	(0.01–0.03)
**Copeptin (pmol/l)**						
median	8.19	7.68	19.6	24.1	24.5	15.0
(IQR)	(4.4–31.4)	(4.2–16.5)	(6.2–61.9)	(8.6–42.4)	(5.2–73.5)	(5.7–62.3)
**Temperature (°C)**						
Mean (±SD)	37.0±0.6	37.0±0.6	36.9±0.7	37.0±0.9	36.8±0.7	37.0±0.7
**Risk factors % (n)**						
Heart failure	13.4 (48/357)	11.6 (34/293)	21.9 (14/64)	25.0 (5/20)	17.4 (4/23)	20.0 (5/25)
AH	80.0 (286/358)	77.7 (227/292)	89.4 (59/66)	85.0 (17/20)	91.7 (22/24)	88.5 (23/26)
PAD	8.3 (30/363)	8.4 (25/298)	7.7 (5/65)	10.0 (2/20)	4.3 (1/23)	7.7 (2/26)
Diabetes mellitus	19.3 (71/367)	18.9 (57/301)	21.2 (14/66)	35.0 (7/20)	25.0 (6/24)	7.7 (2/26)
CHD	21.0 (76/363)	21.2 (63/297)	19.7 (13/66)	25.0 (5/20)	16.7 (4/24)	19.2 (5/26)
Atrial fibrillation	19.4 (69/355)	15.9 (46/289)	34.8 (23/66)	45.0 (9/20)	25.0 (6/24)	38.5 (10/26)
Hyperchol	29.2 (99/339)	29.1 (82/282)	29.8 (17/57)	41.2 (7/17)	25.0 (5/20)	21.7 (5/23)
Family history of stroke	30.1 (106/352)	31.3 (90/288)	25.0 (16/64)	25.0 (5/20)	24.0 (6/25)	21.7 (5/23)
**NIHSS**						
Median	5	4	11	12	9	11
(IQR)	(2–10)	(2–7)	(5–18)	(5–19)	(3–15.5)	(5.5–19)
**Charlson Index**						
Median	1	0	1	1.5	1	0.5
(IQR)	(0–2)	(0–2)	(0–2)	(0–2.5)	(0–2)	(0–2)
**BP on admission**						
**Systolic BP**						
Mean (±SD)	160±29	161±34	158±34	153±36	158±36	159±34
**Diastolic BP**						
Mean (±SD)	86±21	85±20	92±23	103±30	89±22	92±18

UTI: urinary tract infection; CRP: C-reactive protein; WBC: white blood cells; NIHSS: National Institutes of Health Stroke Scale; BP: blood pressure; IQR: interquartile range (log transformed), AH: arterial hypertension; PAD: peripheral artery disease; CHD: coronary heart disease; Hyperchol: Hypercholestrolemia.

### Blood Biomarkers as Predictors of Post-stroke Infections

Copeptin, PCT, WBC and CRP-levels on admission predicted any infection, pneumonia and UTI in the acute phase of stroke. ORs and AUCs for each marker measured on admission (i.e. day 0) are provided in table 2. ORs to predict infections associated with nearest predictor measurements over time (i.e. performed 1 or 2 days prior to the onset of infection) are presented in table 3. After adjusting for either age, NIHSS, CI or infarct localization (infra−/supratentorial) in a bivariate model all biomarkers remained significant predictors (table 4).

**Table 2:** OR/AUC to predict infections (measurements on admission (day 0)).

Univariate analyses variables	OddsRatio	CI (95%)	p-value	AUC
**Any Infection (n = 66)**				
Temperature	0.88	0.59–1.33	0.055	0.51
PCT	1.91	1.38–2.63	<.001	0.68
CRP	1.50	1.22–1.84	<.001	0.65
WBC	3.35	2.14–5.23	<.001	0.74
Mcyt	1.43	1.03–2.00	0.035	0.56
Copeptin	2.51	1.68–3.75	<.001	0.73
**Pneumonia (n = 20)**				
Temperature	0.90	0.48–1.69	0.75	0.49
PCT	1.96	1.34–2.86	<.001	0.69
CRP	1.67	1.25–2.24	<.001	0.77
WBC	3.38	1.85–6.20	<.001	0.76
Mcyt	2.00	1.28–3.11	0.002	0.63
Copeptin	2.35	1.29–4.28	0.005	0.75
**Urinary Tract Infection (n = 25)**				
Temperature	0.77	0.40–1.48	0.43	0.56
PCT	1.90	1.30–2.78	<.001	0.70
CRP	1.61	1.20–2.16	0.002	0.65
WBC	3.23	1.75–5.96	<.001	0.77
Mcyt	1.46	0.89–2.40	0.14	0.54
Copeptin	2.99	1.60–5.60	<.001	0.77
**Other Infection (n = 21)**				
Temperature	0.99	0.48–2.04	0.97	0.46
PCT	1.48	0.96–2.28	0.08	0.66
CRP	1.36	0.96–1.91	0.08	0.60
WBC	4.14	2.13–8.02	<.001	0.78
Mcyt	1.72	1.07–2.76	0.02	0.71
Copeptin	1.70	0.86–3.37	0.13	0.67

OR referred to an increment to predict values from the 1^st^ to the 3^th^ interquartile range (IQR). IQRs for the parameters are given in Table 1.

PCT: procalcitonin; CRP: C-reactive protein; WBC: white blood cells; Mcyt: monocytes.

**Table 3:** Odds ratios/AUC to predict infections associated with nearest predictor measurements*.

Univariate analyses variables	OddsRatio	CI (95%)	p-value	AUC
**Any Infection**				
Temperature	2.30	1.46–3.63	0.0003	0.64
PCT	1.69	1.30–2.20	<.0001	0.67
CRP	2.28	1.75–2.96	<.0001	0.74
WBC	4.91	3.38–7.14	<.0001	0.82
Mcyt	1.72	1.40–2.11	<.0001	0.65
Copeptin	2.40	1.81–3.20	<.0001	0.75
**Pneumonia**				
Temperature	3.00	1.22–7.37	0.02	0.67
PCT	1.95	1.36–2.79	0.0003	0.71
CRP	2.65	1.83–3.84	<.0001	0.80
WBC	4.29	2.52–7.31	<.0001	0.81
Mcyt	2.17	1.71–2.77	<.0001	0.72
Copeptin	3.32	2.32–4.76	<.0001	0.86
**Urinary Tract Infection**				
Temperature	1.64	0.77–3.48	0.20	0.57
PCT	1.61	1.18–2.20	0.003	0.63
CRP	2.26	1.52–3.37	<.0001	0.74
WBC	4.65	2.85–7.58	<.0001	0.83
Mcyt	2.04	1.60–2.60	<.0001	0.69
Copeptin	2.09	1.39–3.13	0.0004	0.71
**Other Infection**				
Temperature	6.82	2.34–19.89	0.0004	0.80
PCT	1.33	0.95–1.85	0.09	0.58
CRP	2.31	1.50–3.55	0.0001	0.74
WBC	5.69	3.44–9.39	<.0001	0.84
Mcyt	1.32	0.93–1.87	0.12	0.61
Copeptin	2.22	1.39–3.55	0.0008	0.75

OR referred to an increment to predict values from the 1^st^ to the 3^th^ interquartile range (IQR). IQRs for the parameters are given in Table 1.

PCT: procalcitonin; CRP: C-reactive protein; WBC: white blood cells; Mcyt: monocytes performed 1 or 2 days prior to the onset of infection.

**Table 4:** OR to predict infections associated with nearest predictor measurements adjusted for age, NIHSS and CI as well as supra- or infratentorial infarct localization.

	OR (95%CI)adjusted for age	OR (95%CI)adjusted for NIHSS	OR (95%CI)adjusted for CI	OR (95%CI) adjusted for supra−/infratentorial infarctions
**Any Infection**				
Temperature	2.36 (1.48–3.75)	2.10 (1.35–3.28)	2.82 (1.46–3.56)	2.30 (1.45–3.65)
PCT	1.64 (1.27–2.12)	1.62 (1.26–2.07)	1.81 (1.37–2.40)	1.69 (1.30–2.20)
CRP	2.23 (1.72–2.90)	1.96 (1.47–2.60)	2.22 (1.70–2.90)	2.28 (1.75–2.96)
WBC	4.97 (3.42–7.21)	4.22 (2.86–6.21)	4.90 (3.34–7.20)	4.80 (3.33–6.91)
Mcyt	1.69 (1.37–2.07)	1.70 (1.37–2.10)	1.68 (1.37–2.06)	1.72 (1.40–2.11)
Copeptin	2.22 (1.64–3.02)	1.84 (1.21–2.79)	2.30 (1.72–3.70)	2.43 (1.81–3.25)
**Pneumonia**				
Temperature	3.11 (1.23–7.86)	2.64 (1.11–6.29)	2.95 (1.23–7.09)	2.95 (1.24–7.00)
PCT	1.89 (1.33–2.67)	1.88 (1.33–2.65)	2.15 (1.40–3.32)	1.95 (1.37–2.79)
CRP	2.58 (1.79–3.71)	2.25 (1.48–3.42)	2.60 (1.77–3.80)	2.67(1.86–3.82)
WBC	4.17 (2.41–7.22)	3.73 (2.17–6.41)	4.32 (2.58–7.23)	4.30 (2.55–7.28)
Mcyt	2.09 (1.63–2.67)	2.13 (1.65–2.75)	2.15 (1.71–2.71)	2.19 (1.72–2.79)
Copeptin	3.07 (2.08–4.53)	2.95 (1.70–5.11)	3.28 (2.24–4.81)	3.37 (2.28–4.98)
**Urinary Tract Infection**				
Temperature	1.66 (0.78–3.55)	1.48 (0.76–2.88)	1.61 (0.76–3.42)	1.63 (0.78–3.42)
PCT	1.56 (1.16–2.10)	1.54 (1.12–2.11)	1.74 (1.19–2.53)	1.67 (1.21–2.29)
CRP	2.21 (1.49–3.29)	1.98 (1.31–3.00)	2.21 (1.45–3.36)	2.46 (1.64–3.69)
WBC	4.50 (2.82–7.18)	4.18 (2.48–7.06)	4.76 (2.75–8.25)	4.86 (2.99–7.92)
Mcyt	1.97 (1.56–2.49)	1.99 (1.56–2.53)	2.02 (1.48–2.77)	2.08 (1.63–2.67)
Copeptin	1.86 (1.20–2.89)	1.65 (0.85–3.20)	1.92 (1.19–3.09)	2.02 (1.32–3.10)
**Other Infection**				
Temperature	6.94 (2.52–19.12)	5.75 (2.10–15.71)	6.57 (2.50–17.29)	6.52 (2.23–19.06)
PCT	1.29 (0.93–1.78)	1.24 (0.87–1.77)	1.37 (0.97–1.92)	1.36 0.99–1.88)
CRP	2.25 (1.50–3.37)	1.91 (1.16–3.14)	2.30 (1.53–3.44)	2.44 (1.56–3.81)
WBC	5.54 (3.49–8.78)	5.01 (2.93–8.56)	5.62 (3.48–9.08)	6.08 (3.75–9.88)
Mcyt	1.32 (0.96–1.82)	1.30 (0.95–1.79)	1.33 (0.95–1.84)	1.34 (0.95–1.91)
Copeptin	2.28 (1.36–3.79)	1.60 (0.75–3.42)	2.37 (1.50–3.74)	2.17 (1.31–3.59)

PCT: procalcitonin; CRP: C-reactive protein; WBC: white blood cells; Mcyt: monocytes.

Copeptin as a new prognostic marker for SAI was a strong predictor of any infection, pneumonia and UTI (table 3). Copeptin had the same prognostic accuracy compared to WBC, CRP, and the only statistical significant difference in AUCs was found when comparing WBC and copeptin regarding the outcome of OI (p = 0.02) (table 5).

**Table 5:** Comparison of AUCs for developing infection between the predictors WBC, Mcyt, CRP and Copeptin.

Variables	AUC	p-value	
**Any Infection**			
WBC vs Mcyt	0.82 vs 0.65	<.001	
WBC vs CRP	0.82 vs 0.74	0.16	
WBC vs Copeptin	0.82 vs 0.75	0.07	
CRP vs Copeptin	0.74 vs 0.75	0.75	
CRP vs Mcyt	0.74 vs 0.65	0.04	
Copeptin vs Mcyt	0.75 vs 0.65	0.05	
**Pneumonia**			
WBC vs Mcyt	0.81 vs 0.72	0.13	
WBC vs CRP	0.81 vs 0.80	0.78	
WBC vs Copeptin	0.81 vs 0.86	0.72	
CRP vs Copeptin	0.80 vs 0.86	0.98	
CRP vs Mcyt	0.80 vs 0.72	0.36	
Copeptin vs Mcyt	0.86 vs 0.72	0.28	
**Urinary Tract Infection**			
WBC vs Mcyt	0.83 vs 0.69	0.09	
WBC vs CRP	0.83 vs 0.74	0.24	
WBC vs Copeptin	0.83 vs 0.71	0.14	
CRP vs Copeptin	0.74 vs 0.71	0.86	
CRP vs Mcyt	0.74 vs 0.69	0.64	
Copeptin vs Mcyt	0.71 vs 0.69	0.68	
**Other Infection**			
WBC vs Mcyt	0.84 vs 0.61	0.008	
WBC vs CRP	0.84 vs 0.74	0.10	
WBC vs Copeptin	0.84 vs 0.75	0.02	
CRP vs Copeptin	0.74 vs 0.75	0.80	
CRP vs Mcyt	0.74 vs 0.61	0.28	
Copeptin vs Mcyt	0.75 vs 0.61	0.30	

PCT: procalcitonin; CRP: C-reactive protein; WBC: white blood cells; Mcyt: monocytes.

### Predictive Models for Post-stroke Infections

We defined two batches of the three parameters with highest AUC values for any infection, pneumonia, UTI and OI by combining WBC, CRP and copeptin (batch 1) as well as WBC, CRP and PCT (batch 2).

Batch 1 (WBC, CRP, copeptin) better predicted any infection (Wald-p<0.001) and pneumonia (Wald-p<0.001) than the best single predictor alone. However, batch 1 was not a better predictor of UTI (Wald-p = 0.058) and OI (Wald-p = 0.25) than WBC (table 6).

**Table 6:** Comparison of batches with best predictors of specific type of infection alone.

	Adjusted OR*	CI (95%)	p-value	AUC	Wald-p**
**Batches 1: WBC+CRP+Copeptin**					
**Any infection**					
WBC	3.70	2.26–6.08	<0.001	0.86	<0.001
CRP	1.66	1.24–2.21	<0.001		
Copeptin	1.53	1.07–2.18	0.019		
**Pneumonia**					
WBC	4.12	1.63–10.39	0.003	0.92	<0.001
CRP	1.92	1.30–2.84	0.001		
Copeptin	2.06	1.19–3.57	0.010		
**Urinary Tract infection**					
WBC	3.11	1.55–6.24	0.001	0.85	0.058
CRP	1.62	1.01–2.61	0.047		
Copeptin	1.26	0.73–2.19	0.411		
**Other Infections**					
WBC	6.84	3.00–15.60	<0.001	0.90	0.43
CRP	1.29	0.87–1.93	0.208		
Copeptin	1.18	0.69–2.03	0.550		
**Batch 2: WBC+CRP+PCT**					
**Any infection**					
WBC	3.67	2.42–5.58	<0.001	0.84	<0.001
CRP	1.56	1.16–2.11	0.003		
PCT	1.25	0.99–1.57	0.064		
**Pneumonia**					
WBC	4.25	2.27–7.97	<0.001	0.90	<0.001
CRP	1.87	1.39–2.52	<0.001		
PCT	1.36	1.00–1.85	0.052		
**Urinary Tract Infection**					
WBC	2.89	1.69–4.95	<0.001	0.82	0.014
CRP	1.59	0.90–2.81	0.114		
PCT	1.19	0.82–1.70	0.359		
**Other Infections**					
WBC	5.74	3.33–9.87	<0.001	0.89	0.25
CRP	1.53	0.92–2.55	0.103		
PCT	0.85	0.59–1.24	0.407		

WBC: white blood cells; CRP: C-reactive protein; PCT: procalcitonin. AUC: Area under the curve to predict infection using the combined model of all predictors.g.

* adjusted for all predictors in the respective model.

** Wald-p: refers to the comparison of the combined model with the model of the strongest predictor, alone which always was WBC.

Batch 2 (WBC, CRP, PCT) better predicted any infection (Wald-p<0.001), pneumonia (Wald-p<0.001) and UTI (Wald-p = 0.014) than the best single predictor alone. However, batch 2 was not better in predicting OI compared to the best single predictor (Wald-p = 0.25) (table 6).

## Discussion

The value of rapidly available blood markers as predictors for SAI has not been studied extensively, although WBC, CRP and Mcyt are routinely measured within the first hours of admission. Copeptin and PCT measured on admission were good predictors of any infection, pneumonia and UTI in the present cohort. They showed a similar predictive value for future infection compared to WBC and CRP. In a recent study neither WBC, CRP, Mcyt nor PCT measured on admission were sensitive enough to reliably be associated with SAI [18]. In another study, WBC and Mcyt count on admission did not differ between infected and non-infected stroke patients [19]. Only on day 1 after stroke onset, body temperature [18] and WBC [18], [19] became significantly associated with infections after stroke. However, in these studies the time point of diagnosis in relation to biomarker measurements was not taken into account. Therefore, they could not really establish the predictive value of these markers but rather their diagnostic accuracy at the time of infection. Moreover the sample size was somewhat small and associations might have been missed due to lack of power. To our knowledge our study is the first to assess the predictive value of these markers taking into account the time point of measurements as well as diagnosis.

In the present study, each laboratory parameter remained a strong predictor after adjusting for NIHSS, age and CI and infarct localization. This is an unexpected finding because age and stroke severity may also contribute to SIS and thus infection after acute ischemic stroke [20]–[22]. However, these biomarkers seem to add prognostic information beyond age, stroke severity and a higher CI as well as infarct localization.

Copeptin was a strong predictor for SAI on admission and during the acute phase of stroke. The predictive value of copeptin in respect of SAI was similar to that of established biomarkers of infection (i.e. WBC, CRP). This finding might be due to the association of copeptin with the activation of the HPAA: increased copeptin-levels probably indicate a high degree of stress and SIS, which means a higher susceptibility to develop an infection. The prognostic value of PCT was also in the range of WBC and CRP. In the literature PCT is a superior diagnostic marker in pneumonia and other bacterial infections when compared to WBC and CRP [23]. However, the prognostic accuracy of a single PCT valueis limited [24]. PCT might be rather a specific than a sensitive prognostic marker in predicting infections.

The combination of established inflammatory makers (WBC, CRP) combined with a biomarker of stress, i.e. copeptin or a biomarker of bacterial infection, i.e. PCT [16] improves prediction of SAI compared to the strongest prognostic marker alone. The combination of biomarkers probably reflects better the complexity of an infection than one biomarker alone and may lead to a more accurate prediction of a beginning but not yet clinically apparent infection.

The investigated biomarkers seem to detect infections before clinical or paraclinical signs prompt further diagnostic work-up leading to the diagnosis of infection. Thus, these markers may help in risk stratification and may select high-risk patients for intervention studies.

We are aware of the following limitations: First, our results are based ona single cohort and our findings need to be validated in an independent and larger cohort. Second, the sample size was relatively small when assessing subgroups of infection. The bivariate analysis may have a limited statistical power and validity underestimating possible effects of biomarkers and other potential predictors. Third, although WBC and CRP was not a criterion for making the diagnosis of pneumonia, any infection and UTI, one must take into account that WBC was one of three criteria for the diagnosis of the subgroup of OI. Therefore, the good predictive value of WBC - in the case of OI - is most probably due to incorporation bias. This, on the other hand, strengthens the predictive value of copeptin that might be underestimated compared to WBC in this study. Fourth, we are not able to proof causalities or provide more insights into pathomechanisms, to explain why these markers are good predictors of infections even before clinical signs occur. But even if these markers are only surrogates of underlying processes which predispose patients for infections, from a clinical standpoint we belief that the observed associations are very interesting since we identified accurate prognostic markers for risk stratification. Finally, the distinction between prediction and early diagnosis of infection is difficult. We are not able to differentiate whether the biomarkers investigated in this study might rather detect infections at an early state or predict vulnerability for future post-stroke infections, although we excluded patients with possible infection prior to the onset of stroke.

In summary, copeptin, PCT, WBC and CRP were good predictors of the development of any infection, pneumonia and UTI. The combination of the 3 biomarkers even improved the prognostic value by accurately separating patients with and without future infections already on admission. If validated in larger prospective studies the combination of these 3 biomarkers with best AUC values may add significant information for the early identification of high-risk patients. Future intervention studies could select patients with high-risk profiles according to these biomarker levels and these high-risk patients may proof to benefit from prophylactic antibiotic treatment.
